# Nitroxyl: A Novel Strategy to Circumvent Diabetes Associated Impairments in Nitric Oxide Signaling

**DOI:** 10.3389/fphar.2020.00727

**Published:** 2020-05-19

**Authors:** Anida Velagic, Chengxue Qin, Owen L. Woodman, John D. Horowitz, Rebecca H. Ritchie, Barbara K. Kemp-Harper

**Affiliations:** ^1^ Heart Failure Pharmacology, Baker Heart and Diabetes Institute, Melbourne, VIC, Australia; ^2^ Central Clinical School, Monash University, Melbourne, VIC, Australia; ^3^ Drug Discovery Biology, Monash Institute of Pharmaceutical Sciences, Monash University, Melbourne, VIC, Australia; ^4^ Basil Hetzel Institute, Queen Elizabeth Hospital, University of Adelaide, Adelaide, SA, Australia; ^5^ Department of Pharmacology, Biomedicine Discovery Institute, Monash University, Melbourne, VIC, Australia

**Keywords:** nitric oxide, diabetes, type 2 diabetes, cardiovascular disease, nitroxyl, HNO, nitric oxide resistance

## Abstract

Diabetes is associated with an increased mortality risk due to cardiovascular complications. Hyperglycemia-induced oxidative stress underlies these complications, leading to an impairment in endogenous nitric oxide (NO•) generation, together with reductions in NO• bioavailability and NO• responsiveness in the vasculature, platelets and myocardium. The latter impairment of responsiveness to NO•, termed NO• resistance, compromises the ability of traditional NO•-based therapeutics to improve hemodynamic status during diabetes-associated cardiovascular emergencies, such as acute myocardial infarction. Whilst a number of agents can ameliorate (e.g. angiotensin converting enzyme [ACE] inhibitors, perhexiline, statins and insulin) or circumvent (e.g. nitrite and sGC activators) NO• resistance, nitroxyl (HNO) donors offer a novel opportunity to circumvent NO• resistance in diabetes. With a suite of vasoprotective properties and an ability to enhance cardiac inotropic and lusitropic responses, coupled with preserved efficacy in the setting of oxidative stress, HNO donors have intact therapeutic potential in the face of diminished NO• signaling. This review explores the major mechanisms by which hyperglycemia-induced oxidative stress drives NO• resistance, and the therapeutic potential of HNO donors to circumvent this to treat cardiovascular complications in type 2 diabetes mellitus.

## Introduction

Globally, over 460 million individuals have diabetes, and this figure is projected to increase to 700 million by the year 2045 (Saeedi et al., 2019). It is estimated that 90% of these individuals have type 2 diabetes mellitus (T2DM), and approximately 10% have type 1 diabetes mellitus (T1DM) (International Diabetes Federation, 2019). The leading cause of morbidity and mortality in individuals with either diabetes subtype is cardiovascular disease (Htay et al., 2019). Individuals with diabetes have an elevated risk of coronary artery disease, peripheral vascular disease, ischemic stroke and heart failure (Almourani et al., 2019). These cardiovascular complications arise largely as a consequence of hyperglycemia-induced oxidative stress, which impairs nitric oxide (NO•) signaling at the level of synthesis and responsiveness (Fiorentino et al., 2013). This loss in NO• responsiveness, termed ‘NO• resistance,' results largely due to “scavenging” of NO• by superoxide and inactivation of its target, soluble guanylyl cyclase (sGC) (Paolocci et al., 2001a; Worthley et al., 2007; Ritchie et al., 2017). NO• resistance affects multiple sites in the cardiovascular system, including the myocardium, vasculature and platelets (Qin et al., 2020). As such, patients with diabetes fail to respond to the anti-aggregatory and vasodilator effects of NO•-based pharmacotherapies during cardiovascular emergencies, such as acute myocardial infarction, transient myocardial ischemia and acute decompensated heart failure (Dautov et al., 2013). Several pharmacotherapies including statins, some angiotensin-converting enzyme (ACE) inhibitors, perhexiline, and insulin (in the presence of severe hyperglycemia) ameliorate NO• resistance (Chirkov and Horowitz, 2007), while sGC activators primarily circumvent the problem (Costell et al., 2012). However, there are limitations associated with their clinical utility, particularly as these amelioration strategies are not instantaneously effective, and thus unsuitable for emergency situations. On the contrary, nitroxyl (HNO) donors circumvent NO• resistance and thus promote vasodilation, while uniquely inducing positive inotropic and lusitropic responses that persist in conditions of oxidative stress (e.g. heart failure, diabetes) where responses to NO• are diminished (Paolocci et al., 2003; Chin et al., 2016; Tare et al., 2017; Qin et al., 2020). Although the aforementioned cardiovascular changes are associated with both T1DM and T2DM, due to the prevalence of the latter, this review will explore the major mechanisms that drive impairments in NO• signaling in T2DM, and highlight the therapeutic potential of HNO donors to circumvent this problem, in order to alleviate acute hemodynamic complications in T2DM.

## Nitric Oxide Signaling in the Cardiovascular System

### Nitric Oxide Synthesis

NO• plays an important role in maintaining cardiovascular homeostasis. This occurs through its vasodilator capacity, inhibition and reversal of platelet aggregation, suppression of inflammation and oxidative stress, inhibition of thrombosis and modulation of vascular smooth muscle cell (VSMC) proliferation and vascular remodeling (Napoli et al., 2006; Ignarro, 2019). NO• is endogenously synthesized by three isoforms of the NO• synthase (NOS) enzyme, specifically, neuronal NOS (nNOS), inducible NOS (iNOS) and endothelial NOS (eNOS), also known as NOS1, NOS2 and NOS3, respectively (Salerno et al., 2018). All three enzymes consist of two subunits, an N-terminal oxygenase domain that binds the substrate L-arginine, cofactor tetrahydrobiopterin (BH_4_) and a heme iron group, and a C-terminal reductase domain that binds nicotinamide adenine dinucleotide phosphate (NADPH), flavin adenine dinucleotide phosphate (FAD) and flavin mononucleotide (FMN) (Qian and Fulton, 2013). Between these domains, exists a calmodulin binding sequence, that binds calcium (Ca^2+^) (Förstermann and Sessa, 2012).

eNOS is considered the predominant isoform constitutively expressed in the cardiovascular system, where it is responsible for the synthesis of NO• in endothelial cells, cardiomyocytes and platelets (Radziwon-Balicka et al., 2017; Ritchie et al., 2017). In endothelial cells, eNOS produces NO• in response to stimulation by shear stress or receptor agonists including bradykinin, acetylcholine, substance P, thrombin, histamine or β-adrenoceptor agonists (Premer et al., 2019). Under basal conditions, eNOS is present in an inactive state bound to either caveolin-1 or caveolin-3, which are located in small invaginations of the plasma membrane known as caveolae, in endothelial cells or cardiomyocytes, respectively (Massion et al., 2003). Upon stimulation by shear stress or agonists, intracellular Ca^2+^ levels increase, leading to recruitment of the Ca^2+^-calmodulin sequence, which displaces caveolin-1 or caveolin-3 from the enzyme leading to eNOS activation (Förstermann and Sessa, 2012). Subsequently, the cofactor BH_4_ and heat shock protein 90 (hsp90) are recruited, together with protein kinase B/Akt, which phosphorylates Ser^1177^, thereby activating eNOS (Sharma et al., 2012). This leads to electron transfer from NADPH by FAD and FMN, allowing O_2_ to bind to the heme iron group on eNOS, resulting in the conversion of L-arginine to NO• and L-citrulline (Stuehr et al., 2004; Mancardi et al., 2005). NO• is also generated from NOS-independent sources such as from nitrite and dietary nitrate. In brief, following absorption of dietary nitrate from the gastrointestinal tract, salivary commensal bacteria reduce nitrate to nitrite. Nitrite can then circulate and be converted to NO• via the nitrite reductase activity of several proteins (e.g. deoxyhemoglobin, xanthine oxidoreductase), providing a NOS-independent pathway for NO• generation (Farah et al., 2018). The major physiological modulator of eNOS activity appears to be tissue concentrations of the competitive NOS antagonist asymmetric dimethylarginine (ADMA) (Böger, 2004; Cooke, 2005).

### Nitric Oxide Signaling in the Vasculature

**Figure 1:**
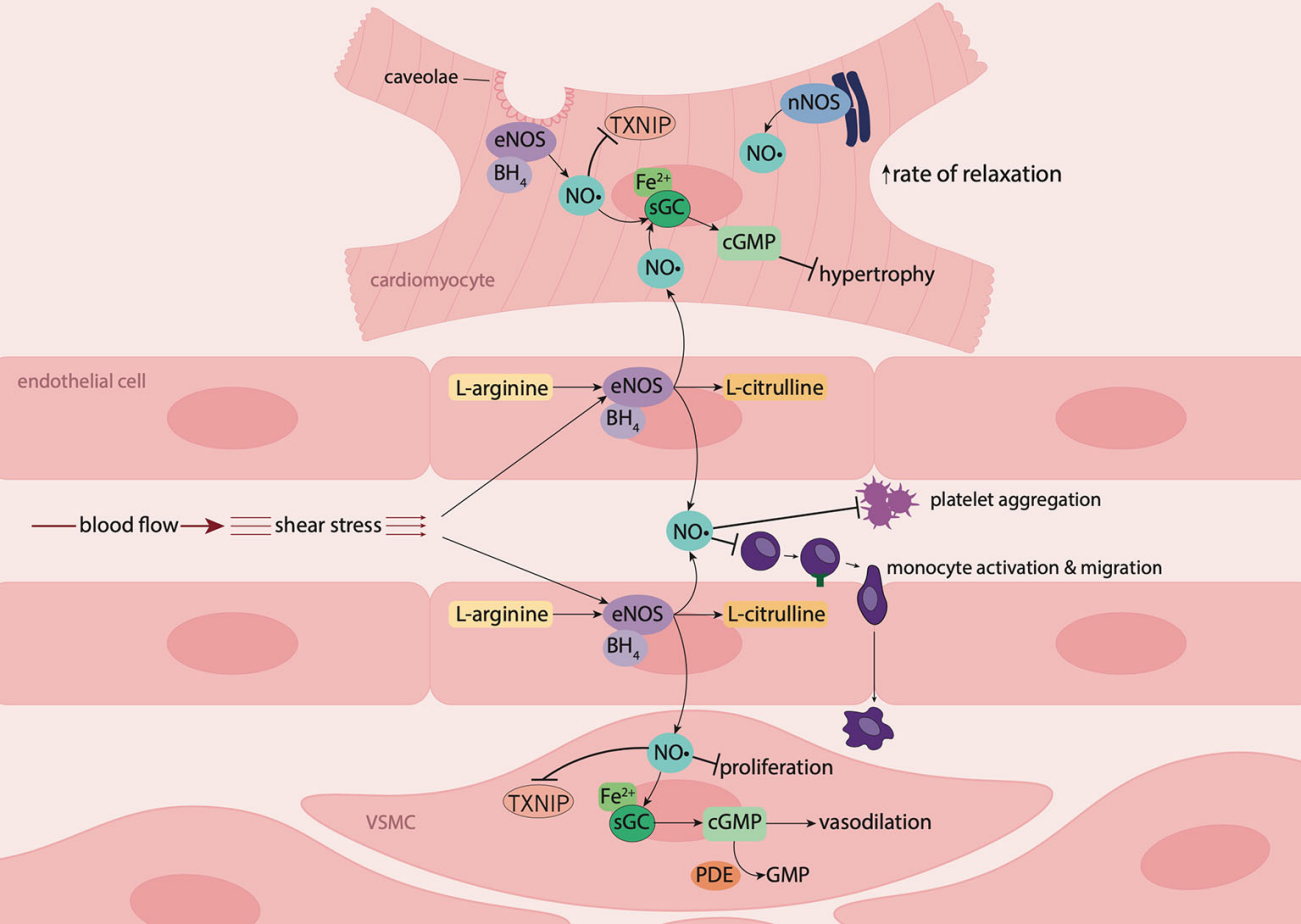
Nitric oxide signaling in the vasculature and myocardium. In endothelial cells and cardiomyocytes, nitric oxide (NO•) is produced by endothelial nitric oxide synthase (eNOS) following stimulation by shear stress (blood flow) or the presence of agonists such as bradykinin. Upon stimulation, the cofactor tetrahydrobiopterin (BH_4_) is recruited, resulting in the conversion of L-arginine to NO• and L-citrulline. In the vascular lumen, NO• inhibits platelet aggregation and leukocyte adhesion and migration. NO• produced by endothelial cells diffuses into underlying vascular smooth muscle cells (VSMCs) where it suppresses proliferation and binds to the ferrous (Fe^2+^) heme group on its biological target, soluble guanylyl cyclase (sGC). Activation of sGC leads to the production of 3',5'-cyclic guanosine monophosphate (cGMP) resulting in vasodilation. NO• produced by endothelial cells from coronary vessels, diffuses into cardiomyocytes, where in combination with NO• produced intracellularly by eNOS and neuronal nitric oxide synthase (nNOS), induces myocardial relaxation, and has anti-hypertrophic effects. NO• also suppresses thioredoxin-interacting protein (TXNIP) formation in cardiomyocytes and the vasculature.

NO• signals predominantly via its intracellular receptor, sGC. In the vasculature, endothelium-derived NO• diffuses into underlying VSMCs in a paracrine manner, where it binds to the ferrous (Fe^2+^) heme iron on sGC (Mancardi et al., 2005; Korkmaz et al., 2018). Activation of sGC leads to the production of 3,5-cyclic guanosine monophosphate (cGMP), intracellular levels of which are regulated by phosphodiesterases (PDEs), which hydrolyze cGMP to GMP (Kass et al., 2007). cGMP effectors include cGMP-dependent protein kinases (cGKs), PDEs and cGMP-gated ion channels (Kemp-Harper and Schmidt, 2009). cGKs phosphorylate target proteins leading to a reduction in intracellular Ca^2+^ concentration, resulting in VSMC relaxation and vasodilation, and suppression of VSMC proliferation (Kemp-Harper and Schmidt, 2009) (**Figure 1**). Similarly, the anti-aggregatory actions of NO• are mediated predominantly via the sGC/cGMP signaling pathway. Thus NO• generated by the endothelium diffuses into the blood vessel lumen where it inhibits platelet aggregation, platelet adhesion to the vascular wall, and thrombosis (Förstermann and Sessa, 2012).

NO• also signals independently of sGC via direct protein S-nitrosylation: NO• reacts with thiols on cysteine residues of target proteins, resulting in modulation of their biological functions (Mancardi et al., 2005; Lima et al., 2010). This is of particular relevance in the vasculature whereby NO• limits superoxide generation via S-nitrosylation of p47^phox^, a critical subunit of the reactive oxygen species (ROS)-generating enzyme, nicotinamide adenine dinucleotide phosphate (NADPH) oxidase 2 (Nox2) (Selemidis et al., 2007), thus protecting against vascular oxidative stress.

Moreover, NO• plays a key anti-inflammatory/anti-atherogenic role, with an ability to limit the activation and transmigration of monocytes through the endothelium to the site of vascular injury via the reduction of endothelial adhesion molecules [e.g. vascular adhesion molecule-1 (VCAM-1); (Spiecker et al., 1997)], chemokine [e.g. monocyte chemoattractant protein-1 (MCP-1); (Zeiher et al., 1995)] and cytokine expression (e.g. interleukin-6 (IL-6); (De Caterina et al., 1995)). These actions of NO• may be explained, in part, by its inhibitory effects on nuclear factor kappa-B (NF-κB) and NLRP3 inflammasome activation. NO• suppresses NF-κB signaling via S-nitrosylation of its regulatory subunit and transcription factor, IKKβ and p65, respectively (Qian and Fulton, 2012). This leads to decreased expression of pro-inflammatory and pro-atherogenic mediators including intracellular adhesion molecule-1 (ICAM-1), VCAM-1 (Spiecker et al., 1997; Qian and Fulton, 2012), MCP-1 and IL-6 (De Caterina et al., 1995; Zeiher et al., 1995). Moreover, NO• regulates the NLRP3 inflammasome, which is a multiprotein signaling complex expressed in macrophages, that activates caspase-1, resulting in the maturation and secretion of the pro-inflammatory cytokines, interleukin-1β (IL-1β) and interleukin-18 (IL-18) (Chen and Sun, 2013). NO• inhibits NLRP3 inflammasome activation, likely via suppression of expression of the NLRP3 activator thioredoxin-interacting protein (TXNIP) (Sverdlov et al., 2013; Chong et al., 2015), thus decreasing caspase-1 activation and secretion of mature IL-1β and IL-18 (Hernandez-Cuellar et al., 2012; Mao et al., 2013). In addition, NO• also has the capacity to limit mast cell degranulation, reducing the release of inflammatory mediators and cytokines and thereby inhibiting the initiation of acute vascular inflammatory processes (Coleman, 2002).

Overall, the entire spectrum of endogenous NO• effects is pro-homeostatic, and confers protection against both atherosclerotic plaque development and rupture. Indeed, NO• normally functions as a ‘firehose', dousing out the flames of incipient plaque rupture and protecting against acute cardiovascular events.

### Nitric Oxide Signaling in the Myocardium

As in the vasculature, NO• plays a key role in the regulation of heart function. NO•, generated by endothelial cells lining the coronary vasculature, diffuses into cardiomyocytes where, in combination with cardiomyocyte nNOS and eNOS-derived NO•, it has anti-hypertrophic effects, enhances myocardial relaxation and improves left ventricular diastolic distensibility (Rosenkranz et al., 2002; Paulus and Bronzwaer, 2004). These actions of NO• are mediated via sGC/cGMP signaling, and are cGK-dependent. cGK modulates excitation-contraction coupling through phosphorylation of troponin I, myosin-binding protein C and titin, thus decreasing myofilament Ca^2+^ sensitivity (Farah et al., 2018). cGK also phosphorylates phospholamban on the sarcoplasmic reticulum Ca^2+^-ATPase pump (SERCA2a) pump, increasing Ca^2+^ reuptake into the sarcoplasmic reticulum (Ritchie et al., 2009). Moreover, cGK suppresses L-type calcium channel activity, further decreasing intracellular Ca^2+^ levels, which attenuates the positive inotropic effects of beta-adrenergic signaling, thus promoting cardiomyocyte relaxation (Massion et al., 2003). cGK also has anti-hypertrophic effects, due to its ability to regulate Ca^2+^ current by modulating L-type Ca^2+^ channel activity, suppress mitogen-activated protein kinases, and inhibit myocyte growth and expression of hypertrophic genes (Ritchie et al., 2009). NO• also modulates myocardial energetic production through its actions on mitochondria, where it inhibits mitochondrial respiration and glucose uptake, and promotes free fatty acid uptake (Massion et al., 2003; Paulus and Bronzwaer, 2004).

## Impaired Cardiovascular Nitric Oxide Signaling in Diabetes

Impaired NO• signaling is present in a wide range of forms of cardiovascular pathologies and has been documented in obesity, diabetes, hypertension, atherosclerosis, congestive cardiac failure, aortic stenosis, angina pectoris, unstable angina, hyperglycemia/diabetes, myocardial infarction, acute atrial fibrillation, ageing and polycystic ovarian syndrome (Lees et al., 1998; Chirkov and Horowitz, 2007; Sverdlov et al., 2014). In the presence of most coronary risk factors, NO• signaling is impacted with evidence of a reduction in both synthesis of and responsiveness to NO• (Worthley et al., 2007; Ito et al., 2015).

### Impaired Nitric Oxide Generation

With regard to NO• generation, severe hyperglycemia can lead to an impairment via reduction of the critical eNOS cofactor BH_4_, increased ADMA, eNOS uncoupling, increased arginase activity and decreased nitrite reduction (Tousoulis et al., 2013; Kövamees et al., 2016). Reduced BH_4_ levels have been identified in human umbilical vein endothelial cells exposed to high glucose conditions, and in aortae isolated from diabetic mice (Xu et al., 2007). In this study, hyperglycemia decreased BH_4_ levels via inhibition of 26S proteasome activity of guanosine 5'-triphosphate cyclohydrolase I (GTPCH), which is a rate-limiting enzyme of BH_4_ synthesis, and increased levels of peroxynitrite, which oxidizes BH_4_ to dihydrobiopterin (BH_2_), thereby uncoupling eNOS (Xu et al., 2007). The authors also found attenuated endothelium-dependent vasodilation in response to acetylcholine in aortae from diabetic mice, indicating impaired vascular generation or responsiveness to NO• per se (Xu et al., 2007). Similarly, increases in forearm blood flow in response to acetylcholine, measured by venous occlusion plethysmography, were impaired in individuals with T2DM, when compared to non-diabetic controls (Heitzer et al., 2000). Interestingly, concomitant infusion of BH_4_ improved forearm blood flow responses to acetylcholine in T2DM, indicating that the observed impairment in endothelium-dependent vasodilation, and hence NO• generation, may be due to decreased BH_4_ (Heitzer et al., 2000).

NOS competes with arginases and arginine methyltransferases (PRMT), for its substrate, L-arginine. Arginase converts L-arginine into L-ornithine or urea, and arginase activity is elevated in disease states associated with endothelial dysfunction, including T2DM (Shemyakin et al., 2012; Kövamees et al., 2016). Similarly, PRMT catalyze the methylation of L-arginine to monomethylarginine (MMA), which is converted to ADMA by type 1 PRMT (Zakrzewicz and Eickelberg, 2009). ADMA is a competitive NOS inhibitor, reducing NO• generation (Vallance et al., 1992). Indeed, plasma ADMA levels have been proposed as a clinically relevant biomarker of endothelial dysfunction and cardiovascular disease (Zhou et al., 2017). Thus, a reduction in endothelium-dependent vasodilation in patients with T2DM, following ingestion of a high fat meal, has been associated with increased levels of ADMA (Fard et al., 2000). However, surprisingly ADMA concentrations are paradoxically lower in diabetic than non-diabetic patients (Horowitz et al., 2018), suggesting that NO• generation by NOS activation is not a major problem in such individuals.

An interesting point for consideration is that endothelial dysfunction has a component of NO• resistance that is difficult to dissect in individual patients by NOS-dependent activation, such as administration of acetylcholine and salbutamol. Thus, it is unclear whether the observed “endothelial dysfunction” is predominantly a reflection of decreased eNOS activity and/or of impaired ability of NO• to signal per se. This distinction between “endothelial dysfunction” and isolated NO• resistance was highlighted in a study by Okon et al., where sensitivity to the endothelium-dependent vasodilator acetylcholine was 10-fold lower in mammary arteries from patients with T2DM, compared to non-diabetic patients, indicating endothelial dysfunction (Okon et al., 2005). Moreover, eNOS gene and protein expression was decreased in T2DM mammary arteries by approximately 50 and 30%, respectively, compared to non-diabetic counterparts (Okon et al., 2005). However, while this reduction in vasodilator response to acetylcholine in T2DM could be due to impaired NO• generation as a consequence of decreased eNOS expression and activity, the authors also found that the vasodilator responses to the endothelium-independent NO• donor, sodium nitroprusside (SNP), were also attenuated in T2DM mammary arteries, indicating reduced vascular responsiveness to NO•, and hence the presence of NO• resistance. Similarly, brachial-artery flow-mediated vasodilation was found to be impaired in patients with T2DM with or without coronary heart disease, when compared to age- and sex-matched non-diabetic controls (Ito et al., 2015). However, the authors did not examine endothelium-independent vasodilation via the use of a NO• donor such as SNP. Therefore, in this study, it is unclear whether the impairment in brachial-artery flow mediated vasodilation in patients with T2DM was due to impaired vascular NO• generation or signaling or both. This highlights the importance of testing vascular responsiveness to a NO• donor to delineate the bases for impaired responses to NOS activators.

### Nitric Oxide Resistance

NO• resistance represents a multifaceted disorder, in which impairments in NO• signaling lead to diminished NO•-responsiveness in platelets, the vasculature and myocardium, resulting in a loss in the vaso- and cardio-protective effects of endogenous and exogenous NO• (Chirkov and Horowitz, 2007). In patients with cardiovascular disease, the presence of NO• resistance is an independent predictor of adverse cardiovascular events and mortality risk (Schachinger et al., 2000; Willoughby et al., 2012). Several studies have identified NO• resistance in T2DM (Williams et al., 1996; van Etten et al., 2002; Anderson et al., 2005; Okon et al., 2005; Shemyakin et al., 2012). In platelets, the NO• donor SNP inhibited aggregation by 15.4 ± 7% in T2DM, compared to 73.1 ± 5.9% in healthy controls, indicating decreased platelet responsiveness to NO• in T2DM, and thus the presence of NO• resistance (Anderson et al., 2005). In studies by Williams et al., and van Etten et al., patients with T2DM displayed vascular NO• resistance, indicated by reduced brachial artery flow-mediated vasodilation in response to SNP, compared to healthy controls (Williams et al., 1996; van Etten et al., 2002). Similarly, vasodilator response of the brachial artery in response to intra-arterial infusion of SNP was lower in patients with coronary artery disease and T2DM, compared to healthy controls, indicating decreased VSMC responsiveness to NO•, and thus NO• resistance (Shemyakin et al., 2012). To the best of our knowledge, the presence of myocardial NO• resistance has not thus far been examined in T2DM. However, in a rat model of T1DM, both myocardial contractile and relaxation responses to the NO• donor diethylamine-NONOate (DEA/NO) were impaired, establishing the presence of myocardial NO• resistance in T1DM (Qin et al., 2020). Thus, it is probable that NO• resistance also occurs at the level of the human myocardium in T2DM, particularly as platelet and vascular responsiveness to NO• is diminished in these patients. Although the pathogenesis of T2DM is different to that of T1DM, they are both characterized by hyperglycemia, and associated with oxidative stress, both of which can contribute to impairment of NO• signaling (Fiorentino et al., 2013).

## Oxidative Stress as a Contributor to Nitric Oxide Resistance

NO• resistance occurs largely due to oxidative stress, where ROS scavenge NO• and reversibly inactivate sGC, resulting in impaired tissue responsiveness to endogenous or exogenous NO• (Dautov et al., 2013; Tare et al., 2017). Oxidative stress refers to an imbalance between the generation of ROS and their clearance by endogenous antioxidants, such as superoxide dismutase, catalase and glutathione peroxidase (Wink et al., 2001). ROS consist of free-radical species, including superoxide, peroxyl, hydroxyl and hydroperoxyl, and non-radicals, such as hydrogen peroxide, peroxynitrite and hypochlorous acid (Phaniendra et al., 2015). They are generated by the mitochondrial electron transport chain, in addition to several other sources including xanthine oxidase, NADPH oxidases, iNOS, and uncoupled eNOS (Brownlee, 2005; Pignatelli et al., 2018).

ROS have a marked impact on NO• generation and signaling. Specifically, NO• reacts rapidly with superoxide forming the powerful oxidant peroxynitrite, which reduces the bioavailability of NO• (Ritchie et al., 2017). In Langendorff-perfused rat hearts, superoxide directly quenches NO•, reducing basal- and agonist-induced NO• release and subsequent vasodilation of the coronary vasculature, in the absence of modifications in eNOS expression or activity (Paolocci et al., 2001a). Peroxynitrite uncouples eNOS by oxidizing BH_4_ to BH_2_, which leads to electron donation to molecular oxygen (O_2_), resulting in the generation of superoxide, further exacerbating oxidative stress (Farah et al., 2018). In addition, peroxynitrite is able to oxidize the ferrous (Fe^2+^) heme group on sGC to its ferric (Fe^3+^) state, desensitizing the enzyme to NO•. Furthermore, oxidation of the heme group weakens its binding, resulting in a heme-free form of sGC which is susceptible to ubiquitin-dependent degradation (Meurer et al., 2009). Consequently, tissue responsiveness to endogenous and exogenous NO• is impaired, resulting in NO• resistance (Ritchie et al., 2017) (**Figure 3**).

### Hyperglycemia-Induced Oxidative Stress

**Figure 2:**
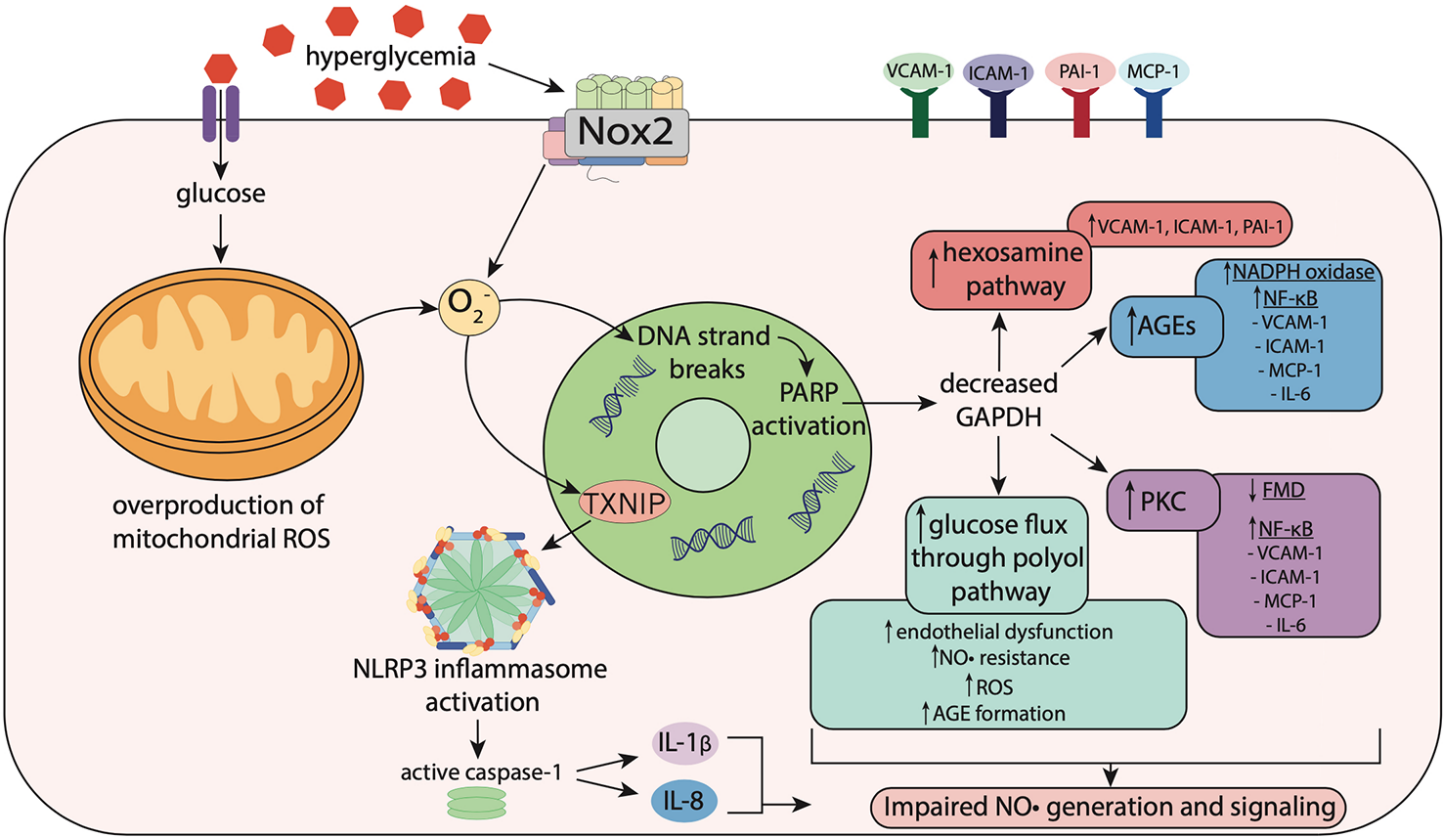
Hyperglycemia-induced oxidative stress impairs nitric oxide signaling. Hyperglycemia induces oxidative stress via increased activity of nicotinamide adenine dinucleotide phosphate (NADPH) oxidase (Nox) enzymes including Nox2, resulting in superoxide (O_2_^−^) generation. In mitochondria, hyperglycemia increases reactive oxygen species (ROS) production including O_2_^−^, which stimulates thioredoxin-interacting protein (TXNIP) expression. TXNIP promotes activation of the NLRP3 inflammasome, which activates caspase-1, resulting in the maturation and secretion of the pro-inflammatory cytokines, interleukin-1β (IL-1β) and interleukin-18 (IL-18). O_2_^−^ also causes strand breaks in DNA, leading to activation of poly (ADP-ribose) polymerase (PARP), which reduces activity of glyceraldehyde-3 phosphate dehydrogenase (GAPDH). Decreased GAPDH activity leads to overactivation of the hexosamine pathway, upregulation of protein kinase C (PKC), elevated glucose flux through the polyol pathway and increased formation of advanced glycation end-products (AGEs). This leads to activation of Nox and NF-κB signaling, resulting in increased expression of pro-inflammatory and pro-atherogenic mediators including vascular adhesion molecule-1 (VCAM-1), intracellular adhesion molecule-1 (ICAM-1), monocyte chemoattractant protein-1 (MCP-1), plasminogen activator inhibitor-1 (PAI-1) and interleukin-6 (IL-6). Upregulation of these pathways results in impaired flow-mediated vasodilation, reflecting both endothelial dysfunction and nitric oxide (NO•) resistance.

In T2DM, hyperglycemia can lead to oxidative stress via increased NADPH oxidase activity, overproduction of mitochondrial ROS and elevated expression of TXNIP (**Figure 2**) (Aon et al., 2015). Elevated myocardial NADPH oxidase-derived superoxide production has been identified in a mouse model of T2DM, and was found to exacerbate left ventricular remodeling and heart failure post-myocardial infarction, when compared to non-diabetic controls (Matsushima et al., 2009). Moreover, internal mammary arteries from patients with T2DM undergoing coronary bypass surgery, displayed elevated NADPH oxidase-derived superoxide levels, and increased membrane translocation of the Nox1/2 regulatory subunits p47^phox^ and Rac1 (Antonopoulos et al., 2015). Nox1 and Nox2 are the major sources of ROS in the vascular wall (Drummond et al., 2011). Therefore, increased membrane translocation of their regulatory subunits p47^phox^ and Rac1 suggests that activation of Nox1/2 is elevated in the vasculature in T2DM.

Hyperglycemia can also stimulate overproduction of mitochondrial ROS, as higher levels of intracellular glucose-derived pyruvate increase flux of electron donors NADH and FADH_2_ into the electron transport chain (Giacco and Brownlee, 2010). This increases the voltage gradient across the mitochondrial membrane, preventing electron transfer within complex III (Giacco and Brownlee, 2010). Consequently, electrons accumulate at coenzyme Q_10_, which donates electrons to molecular oxygen, resulting in superoxide generation, which combined with superoxide generated from other sources such as NADPH oxidase, causes DNA strand breaks (Pacher and Szabó, 2005; Giacco and Brownlee, 2010). This results in activation of poly (ADP-ribose) polymerase (PARP), an enzyme involved in DNA repair, which under normal conditions, resides within the nucleus in an inactive state (Brownlee, 2005). Once activated, PARP modifies the glycolytic enzyme glyceraldehyde-3 phosphate dehydrogenase (GAPDH), reducing its activity (Brownlee, 2005). Decreased GAPDH activity leads to overactivation of the hexosamine pathway, upregulation of protein kinase C (PKC), elevated glucose flux through the polyol pathway and increased formation of advanced glycation end-products (AGEs), all of which can promote impairments in NO• generation and/or signaling (Fiorentino et al., 2013) (**Figure 2**).

#### Hexosamine Pathway

In the hexosamine pathway, O-GlcNAc transferase (OGT) catalyzes a post-translational modification, known as O-GlcNAcylation, of proteins modulating their biological activity (Qin et al., 2017). Increased O-GlcNAcylation has been identified in patients with T2DM (Degrell et al., 2009; Springhorn et al., 2012). Overactivation of this pathway can lead to elevated endothelial expression of the pro-inflammatory and pro-atherogenic mediators VCAM-1, ICAM-1 and plasminogen activator inhibitor-1 (PAI-1) (Fiorentino et al., 2013) (**Figure 2**). A recent study identified decreased NO• production, reduced eNOS expression, and increased O-GlcNAcylation of eNOS in perivascular adipose tissue from thoracic aortae from rats with metabolic syndrome (da Costa et al., 2018). As a result, the anti-contractile capacity of the perivascular adipose tissue was diminished (da Costa et al., 2018). Moreover, increased O-GlcNAcylation of myocardial proteins has been associated with cardiac dysfunction in mouse models of T2DM (Fülöp et al., 2007; Marsh et al., 2011).

#### Protein Kinase C

PKC consists of a family of serine/threonine kinases, which play an important role in signal transduction of several vascular functions, including regulation of angiogenesis, endothelial cell permeability, extracellular matrix deposition and vasoconstriction (Kizub et al., 2014). In a study by Tabit et al., in patients with T2DM, endothelial expression of PKCβ was elevated and associated with impaired flow-mediated dilation of the brachial artery (Tabit et al., 2013). The authors also found increased endothelial levels of the peroxynitrite derivative nitrotyrosine, and elevated activity of NF-κB, indicating the presence of endothelial inflammation and nitrosative/oxidative stress in T2DM (Tabit et al., 2013).

#### The Polyol Pathway

In the polyol pathway, glucose is reduced to sorbitol by aldose reductase, which consumes NADPH in the process (Chandra et al., 2002). NADPH consumption limits NO• production and exacerbates oxidative stress, as NADPH is required for the production of NO• and the antioxidant reduced glutathione (Ramana et al., 2003; Lorenzi, 2007). The deleterious effects of sorbitol on the microvasculature have been demonstrated in rat gracilis arterioles, where exposure to sorbitol stimulated vascular production of ROS, and impaired endothelium-dependent and endothelium-independent vasodilation to a NO• donor, indicating that sorbitol can induce endothelial dysfunction with a substantial component of NO• resistance (Toth et al., 2007) (**Figure 2**). In addition, methylglyoxal, an intermediate of this pathway, is implicated in endothelial dysfunction and elevated in patients with T2DM (Wang et al., 2007; Lund et al., 2011). Methylglyoxal is also a potent glycating agent that reacts with intracellular and extracellular proteins, resulting in the formation of AGEs (Lund et al., 2011).

#### Advanced Glycation End-Products

AGEs are formed during non-enzymatic reactions between reducing sugars or sugar-derived products and amino groups on proteins, lipids or nucleic acids (Barlovic et al., 2010). AGEs and their receptor (receptor for AGEs; RAGE) play a central role in the pathogenesis of vascular complications (Thomas et al., 2005). In patients with T2DM, plasma levels of AGEs are elevated, and negatively correlated with endothelium-dependent and endothelium-independent vasodilation (Tan et al., 2002). Moreover, plasma levels of AGEs are approximately 74% higher in patients with T2DM with vascular complications, compared to those with T2DM without vascular complications (Farhan and Hussain, 2019). Activation of RAGE by AGEs also stimulates NADPH oxidase, increasing intracellular ROS generation, subsequently activating NF-κB, resulting in the production of pro-inflammatory and pro-atherogenic mediators, including IL-6, VCAM-1, ICAM-1 and MCP-1 (Barlovic et al., 2010) (**Figure 2**). Consequently, oxidative stress and inflammation are exacerbated, leading to further impairments in NO• signaling (Andrews et al., 2016). NF-κB activation also stimulates NO• generation by iNOS, however, when superoxide levels are elevated, superoxide anions react with NO• forming peroxynitrite (Wright et al., 2006). This leads to a loss of the inhibitory effects of iNOS-derived NO• on mast cell stabilization (Coleman, 2002), which is critical for prevention of plaque rupture and coronary vasospasm, which precede most ischemic emergencies (Kovanen et al., 1995; Laine et al., 1999).

#### Thioredoxin-Interacting Protein (TXNIP)

Hyperglycemia is also associated with increased expression of TXNIP via the glucose-response element in its gene (Turturro et al., 2007). Thioredoxin (TRX) is a key modulator of intracellular redox stress, possessing antioxidant activity. TXNIP serves as a negative regulator of TRX and contributes to oxidative stress in diabetes, in addition to promoting inflammation with a key role in NLRP3 inflammasome activation (Schulze et al., 2004; Zhou et al., 2010) (**Figure 2**). TXNIP gene expression is elevated in peripheral bone mononuclear cells from patients with T2DM, compared to patients with T1DM and non-diabetic controls (Szpigel et al., 2018). This increase in TXNIP was accompanied by elevated gene expression of NLRP3 and IL-1β, indicating that TXNIP promotes inflammation in T2DM through activation of the NLRP3 inflammasome (Szpigel et al., 2018). These actions of TXNIP are likely to negatively impact NO• signaling in T2DM. Indeed, a reciprocal relationship between platelet NO• responsiveness and TXNIP expression has been demonstrated (Sverdlov et al., 2013).

Collectively, these data provide robust evidence for a key role of oxidative stress and inflammation in the development of cardiovascular complications in T2DM. Central to the disease pathology is an impairment in endogenous and exogenous NO• signaling. Taking into consideration the more prominent role of NO• resistance in impaired NO• signaling in diabetes, there is a strong therapeutic focus on overcoming NO• resistance in this disease setting.

## Current and Emerging Pharmacotherapies to Overcome Impaired Nitric Oxide Signaling: A Focus on Nitric Oxide Resistance

As highlighted, NO• resistance is particularly debilitating in T2DM, where the mortality risk associated with cardiovascular emergencies is increased (e.g. acute myocardial infarction, transient myocardial ischemia, acute pulmonary edema) (Lindholm et al., 2005). In these circumstances, rapid vasodilator and anti-aggregatory actions are required yet NO•-based pharmacotherapies are ineffective. Thus, whilst nitrovasodilators, including the organic nitrate glyceryl trinitrate (GTN), have been clinically utilized since 1876 for the treatment of angina pectoris and heart failure (Sun et al., 2011), their effectiveness is diminished in the very conditions for which they are most needed. As such, NO•-independent therapeutic approaches aimed at ameliorating or circumventing NO• resistance, particularly to manage cardiovascular emergencies, are urgently required for the diabetic population.

### Amelioration Strategies

A number of treatments, including some ACE inhibitors, perhexiline, statins, and reversal of severe hyperglycemia have been shown to ameliorate NO• resistance (**Table 1**).

**Table 1:** Current and emerging therapies to ameliorate and circumvent nitric oxide resistance.

Therapy	Properties	Limitations	References
*Amelioration strategies*			
**ACE inhibitors:** **Ramipril & Perindopril**	•Decrease angiotensin II formation •Improve endothelial function by decreasing bradykinin degradation	•Benefits observed following prolonged use (days to months) •Limited utility during cardiovascular emergencies	(Murphey et al., 2003; Chirkov et al., 2004; Lob et al., 2006; Willoughby et al., 2012)
**Perhexiline**	•Anti-ischemic •Inhibits mitochondrial enzyme carnitine palmitoyltransferase	•Potential neuro- & hepato-toxicity •Variable pharmacokinetics: close therapeutic monitoring required	(Ashrafian et al., 2007; Chong et al., 2016)
**Statins**	•Lower cholesterol •Increase hepatic LDL uptake •Enhance eNOS gene expression •Enhance eNOS activity by reducing caveolin-1 expression	•Benefits observed following prolonged use (days to months) •Limited utility during cardiovascular emergencies	(Willoughby et al., 2002; Stepien et al., 2003; Chirkov et al., 2004; Lundberg et al., 2015; Go et al., 2019)
**Insulin** **(in presence of severe hyperglycemia)**	•Lower plasma glucose •Reduce oxidative stress and superoxide production	•NO• resistance can persist following acute, aggressive glycaemic control •Beneficial effects on mortality unclear	(Vehkavaara and Yki-Järvinen, 2004; Mehta et al., 2005; Worthley et al., 2007)
*Circumvention strategies*			
**sGC activators**	•Bind to heme pocket of sGC •Heme-independent •Activate sGC if heme is oxidized or detached	•Can cause hypotension	(Follmann et al., 2013; Buys et al., 2018; Elgert et al., 2019)
**Nitrite**	•Converted to NO• via reductases •Vasodilator & anti-aggregatory actions potentiated in hypoxia	•Anti-platelet effect diminished in patients with IHD •Does not reduce infarct size post-acute myocardial infarction	(Dautov et al., 2013; Siddiqi et al., 2014; Jones et al., 2015)
**Nitroxyl donors**	•Vasodilator, anti-aggregatory, positive cardiac inotropic/lusitropic actions •sGC-dependent & -independent signaling •Resistant to oxidative stress	•May cause coronary steal •Long-term benefits remain to be elucidated	(Irvine et al., 2007; Kemp-Harper, 2011; Dautov et al., 2013)

#### ACE Inhibitors

Increased activity of the renin-angiotensin system and the generation of angiotensin II, which possesses pro-oxidative, pro-inflammatory and vasoconstrictive properties, is associated with many cardiovascular diseases (Ruszkowski et al., 2019). The ACE inhibitors ramipril and perindopril decrease the formation of angiotensin II, and in large clinical trials (HOPE, Heart Outcomes Prevention Evaluation; EUROPA, European Trial on Reduction of Cardiac Events with Perindopril), have been shown to reduce the incidence of myocardial infarction, cardiac arrest, heart failure, stroke and diabetes-related complications in aging, high-risk adults with vascular disease or diabetes (Yusuf et al., 2000; Fox, 2003).

Whilst the precise mechanisms underlying the improved cardiovascular outcomes following treatment with ramipril or perindopril remain unclear, an ability of these ACE inhibitors to ameliorate NO• resistance may contribute to treatment benefit. Ramipril and perindopril have been shown to improve NO• donor responsiveness, at least at the level of the platelet. In individuals with chronic heart failure, treatment with perindopril for 4 days improved platelet responsiveness to SNP, reducing the proportion of subjects with platelet NO• resistance from 40 to 0% (Chirkov et al., 2004). Similarly, the effect of ramipril on platelet responsiveness to SNP was assessed in a randomized, placebo-controlled, blinded study in older adults (aged ≥50 years) of high cardiovascular risk (history of stroke, coronary artery disease, peripheral vascular disease and/or diabetes) (Willoughby et al., 2012). In this study, 3 months of ramipril therapy decreased systolic and diastolic pressure, and reduced augmentation index (Aix) and plasma levels of ADMA, markers of arterial stiffness and endothelial dysfunction, respectively (Willoughby et al., 2012). The authors also identified a separate group of participants with diabetes, who displayed severe platelet NO• resistance at baseline, in which ramipril therapy for 2 weeks improved platelet responsiveness to SNP, suggesting improved sensitivity of sGC to NO• (Willoughby et al., 2012).

Based on these findings, it is clear that long-term therapy with the ACE inhibitors ramipril or perindopril overcomes NO• resistance and provides protection against adverse cardiovascular events in high-risk populations. However, ACE inhibitor-mediated reversal of NO• resistance is not an option for patients who are intolerant of ACE inhibition, many of whom are diabetic (Neutel, 2010; DuMond and King, 2011). Moreover, the use of ACE inhibitors to attenuate NO• resistance in a cardiovascular emergency is clinically impracticable with the CONSENSUS II study (Cooperative New Scandinavian Enalapril Survival Study II) showing that the intravenous administration of the ACE inhibitor, enalaprilat, within 24 h of an acute myocardial infarct, increased mortality (Swedberg et al., 1992).

#### Perhexiline

The anti-anginal agent, perhexiline, has shown some promise with regard to ameliorating NO• resistance. Used in patients refractory to commonly used antianginal therapies, the anti-ischemic properties of perhexiline are attributed to its ability to inhibit the mitochondrial enzyme carnitine palmitoyltransferase, leading to the reduction in fatty acid metabolism and a shift to greater carbohydrate metabolism by the myocardium (oxygen sparing effect) (Ashrafian et al., 2007). In addition, perhexiline limits oxidative stress via inhibition of NADPH oxidase (Kennedy et al., 2006). Studies have demonstrated an ability of perhexiline to improve platelet SNP responsiveness in patients with stable angina pectoris (Chirkov et al., 2001), acute coronary syndromes (Willoughby et al., 2002) and aortic stenosis (Chirkov et al., 2002). However, the clinical utility of perhexiline is limited due to its complex pharmacokinetics and potential to cause hepatic- and neuro-toxicity, necessitating close therapeutic monitoring (Chong et al., 2016).

#### Statins

Statins are lipid-lowering drugs that are primarily used to treat hypercholesterolemia and prevent the progression of cardiovascular disease (Ko et al., 2019). Whilst statin therapy is associated with improvement in endothelial function in patients with cardiovascular disease (Reriani et al., 2011), evidence in support of an ability of statins to ameliorate vascular NO• resistance is conflicting. Thus, in patients with T1DM, atorvastatin (40 mg/day; 6 weeks) was shown to improve nitroglycerin-mediated dilatation in the brachial artery (Dogra et al., 2005). Similarly, following an acute coronary syndrome, atorvastatin treatment (80 mg/day, 16 weeks) lead to an improvement in GTN-mediated dilation (Dupuis et al., 2005). By contrast, atorvastatin (40 mg/day; 6 weeks) therapy in patients with non-ischemic chronic heart failure, did not lead to an improvement in GTN-mediated dilation in the brachial artery (Strey et al., 2006). At the level of the platelet there is data, albeit limited, to suggest that statin therapy ameliorates NO• resistance. For example, in patients with acute coronary syndrome, pharmacotherapy with statins was associated with improved anti-aggregatory actions of SNP (Chirkov et al., 2001). In addition, in individuals with mild hypercholesterolemia, treatment with pravastatin (40 mg/day) for 3 months, improved inhibition of platelet aggregation in response to SNP (Stepien et al., 2003). Of note, the ability of statins to ameliorate NO• resistance is unlikely due to their cholesterol lowering actions per se, rather their pleiotropic effects such as an ability to reduce superoxide generation and oxidative stress may be responsible. However, acute introduction of statins during evolving acute myocardial infarction has not convincingly improved outcomes (Ostadal, 2012; Vavuranakis et al., 2017).

#### Reversal of Severe Hyperglycemia

As discussed, hyperglycemia is a key contributor to the endothelial dysfunction and macrovascular complications associated with diabetes. Moreover, hyperglycemia contributes to vascular NO• resistance, such that glucose lowering, with long-term insulin treatment (3.5 years), improves brachial artery vasodilatation to SNP in patients with T2DM (Vehkavaara and Yki-Järvinen, 2004). Similarly, in diabetic patients with severe hyperglycemia and acute coronary syndromes, rapid correction of hyperglycemia via intravenous insulin (12 h) increases platelet responsiveness to NO• (Worthley et al., 2007). Such protective actions of insulin are likely due to its ability to reduce oxidative stress, independently of the potential for acute modulation of platelet TXNIP expression (Chong et al., 2015). Indeed, in the DIGAMI study (﻿Diabetes Mellitus Insulin-Glucose Infusion in Acute Myocardial Infarction), insulin infusion post-acute myocardial infarction, followed by multi-dose subcutaneous insulin administration, decreased mortality rate in patients with diabetes (Malmberg et al., 1995). These findings suggest that the relatively rapid effects of infused insulin on platelet NO• responsiveness are of use in patients experiencing acute myocardial infarction, however, it should be noted that a component of NO• resistance may persist following acute, aggressive glycemic control (12 h insulin i.v. infusion) (Worthley et al., 2007).

Whilst the pharmacotherapies discussed have the potential to ameliorate NO• resistance at the level of the vasculature and platelet, all of these strategies have delayed onset of activity, taking hours to days to take effect. As such they are unsuitable for emergency situations (e.g. acute MI, transient myocardial ischemia or acute pulmonary edema), in which rapid circumvention of NO• resistance is required. This is of particular relevance in the diabetic population in which cardiovascular emergencies occur with greater frequency.

### Circumvention Strategies

Emerging therapeutic strategies to circumvent NO• resistance, include sGC activators, nitrite and HNO donors (**Table 1**).

#### sGC Activators

Since NO• resistance is associated with sGC oxidation to the NO•-insensitive Fe^3+^-sGC and subsequent heme-deplete sGC forms, the use of NO•- and heme-independent sGC activators to overcome this limitation has gained considerable attention. sGC activators target sGC in its oxidized (Fe^3+^) or heme-free states and as such have greater efficacy under conditions of oxidative stress (Sandner et al., 2019). Pre-clinical studies have identified protective effects of sGC activators against ischemia/reperfusion injury, myocardial infarction, diabetic cardiomyopathy and diabetic nephropathy (Mátyás et al., 2015; Boustany-Kari et al., 2016; Lee et al., 2017). Furthermore, sGC activators may ameliorate NO• resistance. Thus, chronic treatment of rats with heart failure with the sGC activator, ataciguat (10 mg/kg/twice daily, 10 weeks) improved the vascular response to exogenous NO• in aortic rings and reduced platelet activation (Schäfer et al., 2010). Importantly, sGC activators themselves are anti-aggregatory agents and their anti-platelet actions in both humans (Mendes-Silverio et al., 2012) and rodents (Roger et al., 2010) are augmented when sGC is oxidized. Such findings suggest that sGC activators have the potential to circumvent NO• resistance. Currently, however, the clinical utility of this class of compound is unclear given their profound, and sustained, blood pressure lowering effects in patients with acute decompensated heart failure (Breitenstein et al., 2017) and peripheral arterial occlusive disease (Sandner et al., 2019), in the absence of a clear therapeutic benefit.

#### Nitrite

There is growing interest in the therapeutic potential of nitrite, both as an alternate source of NO• and a signaling molecule in its own right. Importantly, the vasodilator and anti-aggregatory responses to nitrite are potentiated in the setting of hypoxia, suggesting that it may have considerable advantages in the treatment of acute cardiovascular disorders (Dautov et al., 2014). However, current evidence in support of an ability of nitrite to circumvent NO• resistance is limited, and hinges on the concept that part of the effects of nitrite are NO•- and sGC-independent. Thus, in platelets from patients with ischemic heart disease, in whom NO• resistance is evident, the anti-aggregatory effects of nitrite were found also to be diminished (Dautov et al., 2014). By contrast, in heart failure patients with preserved ejection fraction (HFpEF), resistance to the anti-aggregatory actions of SNP was evident, yet the ability of nitrite to inhibit platelet aggregation was maintained (Borgognone et al., 2018). Whilst these anti-aggregatory actions of nitrite were mediated via sGC activation, they were only due, in part, to NO•. The clinical utility of nitrite in the circumvention of NO• resistance is also tempered by the finding that in two clinical trials in patients with acute myocardial infarction (NIAMI and NITRITE-AMI), nitrite administration prior to reperfusion, did not reduce infarct size (Siddiqi et al., 2014; Jones et al., 2015).

#### Nitroxyl-Based Therapies

**Figure 3:**
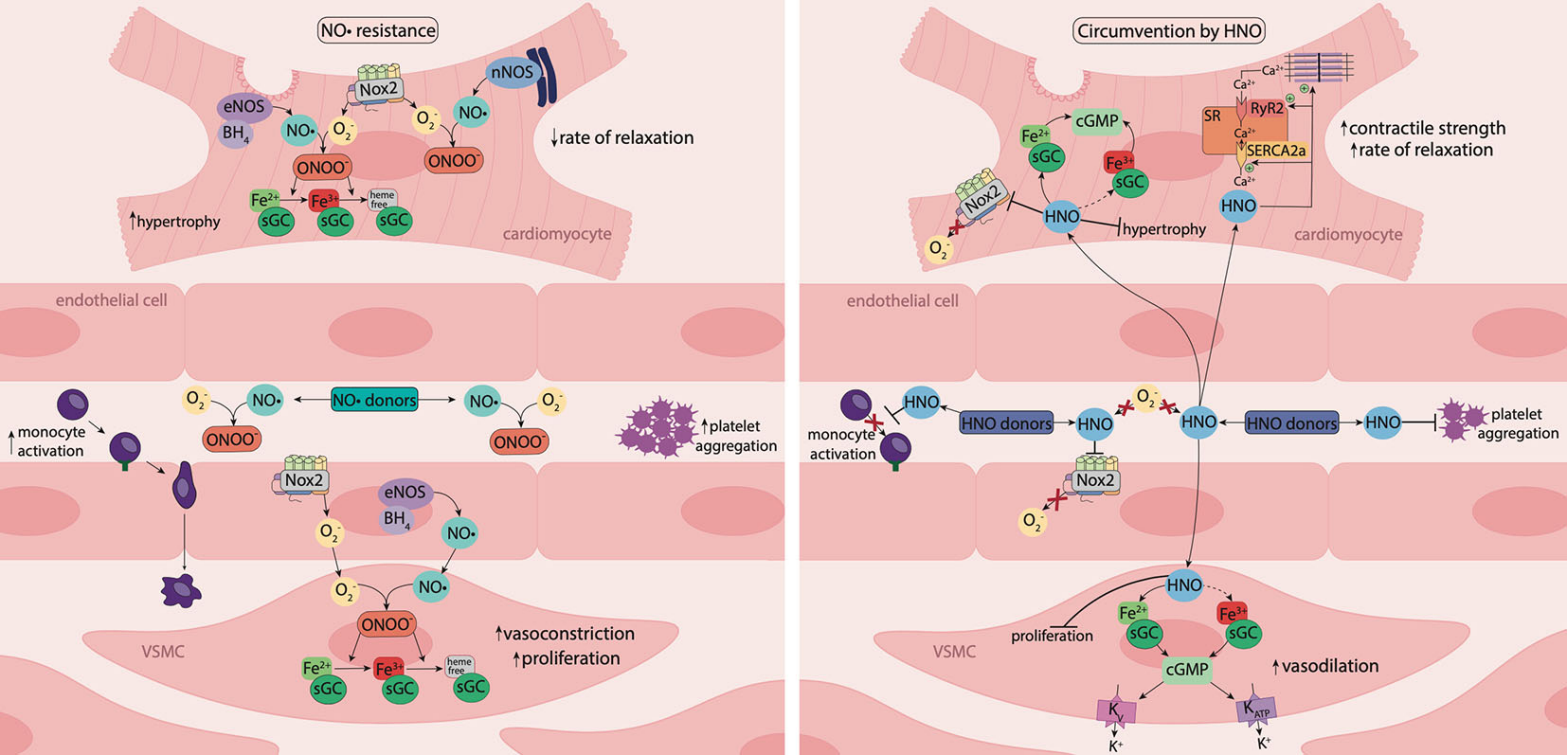
Nitric oxide resistance and its circumvention by nitroxyl. Under oxidative stress, activity of nicotinamide adenine dinucleotide phosphate (NADPH) oxidase (Nox) enzymes, such as Nox2, is elevated, resulting in increased superoxide (O_2_^−^) generation. O_2_^−^ reacts with nitric oxide (NO•), generating the powerful oxidant, peroxynitrite (ONOO^-^), which oxidizes the ferrous (Fe^2+^) heme group of sGC to the ferric (Fe^3+^) or heme-free state, desensitizing the enzyme to NO•. Consequently, tissue responsiveness to NO• is impaired, resulting in NO• resistance. Nitroxyl (HNO) is resistant to scavenging by O_2_^−^ and HNO donors offer an opportunity to circumvent NO• resistance. In the vasculature, HNO causes vasorelaxation, inhibits platelet aggregation and reduces monocyte activation. In vascular smooth muscle cells (VSMCs), HNO signals predominantly via activation of sGC and the subsequent increase in 3',5'-cyclic guanosine monophosphate (cGMP) and may activate the oxidized (Fe^3+^) form of sGC. HNO also targets vascular voltage-dependent and ATP-sensitive K^+^ channels through a cGMP-dependent mechanism. In the vasculature and myocardium, HNO interacts directly with Nox2 to suppresses O_2_^-^ generation. In cardiomyocytes, HNO has anti-hypertrophic effects, and reacts directly with thiols and thiol-containing proteins including the sarcoplasmic reticulum Ca^2+^-ATPase pump (SERCA2a) and ryanodine receptors (RyR2) to enhance Ca^2+^ cycling, together with increasing myofilament Ca^2+^ sensitivity, resulting in enhanced myocardial contractility and relaxation. The vaso- and cardio-protective actions of HNO are preserved in the setting of oxidative stress and HNO donors offer a new therapeutic approach to treat diabetes-associated cardiovascular complications.

HNO is the one-electron reduced and protonated form of NO•. Using the prototypical HNO donors Angeli's salt (which is also a source of nitrite) and iso-propylamine-NONOate (IPA-NO), HNO has been shown to have distinct pharmacological properties and therapeutic advantages when compared to its redox sibling (Irvine et al., 2008; Bullen et al., 2011a). Specifically, unlike NO•, the actions of HNO are preserved during oxidative stress, as HNO is resistant to scavenging by superoxide (Irvine et al., 2007; Kemp-Harper et al., 2016). Moreover, HNO causes venous and arterial dilation (Tare et al., 2017), suppresses vascular generation of ROS via rapid (within minutes), and direct, inhibition of Nox2 oxidase (Miller et al., 2013) and is resistant to tolerance development (unlike organic nitrates) (Irvine et al., 2007). HNO also inhibits VSMC proliferation and platelet aggregation (Tsihlis et al., 2010; Bullen et al., 2011b; Dautov et al., 2013), and reduces endothelial expression of adhesion molecules, monocyte activation and leukocyte adhesion (Andrews et al., 2016). In the vasculature, HNO induces vasodilation predominantly through sGC/cGMP signaling and the preference of HNO for ferric (Fe^3+^) versus ferrous (Fe^2+^) heme groups (Miranda et al., 2003) raises the interesting possibility that HNO may preferentially target the oxidized (Fe^3+^) versus reduced (Fe^2+^) forms of sGC. Such a property may contribute to the preserved efficacy of HNO in the face of oxidative stress, yet studies to date have not provided evidence in support of an ability of HNO to activate oxidized sGC (Miller et al., 2009; Zeller et al., 2009). In comparison to NO•, HNO can also signal via distinct vascular pathways, including the activation of voltage-dependent K^+^ channels (Irvine et al., 2003; Andrews et al., 2009), ATP-sensitive K^+^ channels and the release of calcitonin gene-related peptide (**Figure 3**) (Favaloro and Kemp-Harper, 2007; Chin et al., 2014). Interestingly, HNO might also be endogenously generated, however, in the absence of a validated method to measure tissue levels, this is yet to be established conclusively (Andrews et al., 2009; Kahlberg et al., 2016; Fukuto, 2019). Another unique feature of HNO in comparison to NO•, is the ability of HNO to react directly with thiols and thiol-containing proteins (i.e. cysteines), independently of sGC/cGMP signaling (Kemp-Harper, 2011). In the myocardium, this property allows HNO to serve as a positive cardiac inotrope, interacting with cysteine residues on thiol-containing proteins including ryanodine receptors (Tocchetti et al., 2007) and phospholamban, the regulatory phosphoprotein of the sarcoplasmic reticulum Ca^2+^-ATPase pump (SERCA2a), to enhance Ca^2+^ cycling (Keceli et al., 2019). HNO also increases myofilament calcium sensitivity by promoting the formation of disulfide bonds between myofilament cysteine residues (Gao et al., 2012), Together, these actions of HNO result in enhanced myocardial contractility and relaxation (**Figure 3**) (Paolocci et al., 2007; Tocchetti et al., 2007). It should also be noted that differently from legacy inotropes, the inotropic response to HNO does not require the entry of extracellular Ca^2+^ (Kohr et al., 2010).

These unique properties of HNO, together with its preserved efficacy in the setting of oxidative stress, suggest that HNO donors may circumvent NO• resistance and be of clinical utility in a cardiovascular emergency.

Indeed, HNO donor compounds have displayed vaso- and cardio-protective efficacy, particularly in disease states where endothelial dysfunction and NO• resistance is present (Andrews et al., 2016). At the level of the vasculature, there is evidence to support an ability of both endogenous and exogenous HNO to circumvent NO• resistance. Thus, in the diabetic rat aorta (Leo et al., 2012) and small mesenteric arteries (Kahlberg et al., 2016; Tare et al., 2017), endothelium-dependent relaxation mediated by HNO is preserved, yet that mediated by NO• is impaired. Moreover, in pre-clinical studies, vasorelaxation to the HNO donors, Angeli's salt and IPA/NO are maintained in the diabetic (Leo et al., 2012; Tare et al., 2017), hypercholesterolemic (Bullen et al., 2011b) and hypertensive (Wynne et al., 2012; Irvine et al., 2013) vasculature. Importantly, HNO can also circumvent platelet NO• resistance. Thus, in hypercholesterolemic mice we have shown that the anti-aggregatory actions of the HNO donor, IPA/NO are preserved, yet those to the NO• donor, GTN are impaired (Bullen et al., 2011b). Moreover, in patients with coronary artery disease, the impaired anti-platelet response to the NO• donor, SNP, is circumvented by the HNO donor, IPA/NO (Dautov et al., 2013).

Whilst the discussion so far has focused on circumventing NO• resistance in the vasculature and platelets, it is pertinent to note that the myocardium is also susceptible to NO• resistance, such that NO• can no longer enhance left ventricular (LV) relaxation (Qin et al., 2020). Thus, following ischemia–reperfusion (I–R) injury (Chin et al., 2016) or the induction of T1DM (Qin et al., 2020) in rats, the ability of the NO• donor, DEA/NO to enhance myocardial relaxation is impaired. Such an impairment in myocardial responsiveness to NO• (endogenous or exogenous) may facilitate LV dysfunction, LV hypertrophy and cardiac remodelling (Nightingale et al., 2011; Sverdlov et al., 2011). Recent pre-clinical studies have demonstrated that HNO donors can circumvent myocardial NO• resistance. Specifically, unlike DEA/NO, myocardial relaxation to Angeli's salt is preserved following I–R injury in rat isolated hearts (Chin et al., 2016). In addition, Angeli's salt was found to reduce the duration of ventricular fibrillation following I–R injury (Chin et al., 2016). Similarly, the HNO donor IPA/NO enhanced myocardial relaxation and contraction responses in diabetic rat hearts, while responses to DEA/NO were attenuated (Qin et al., 2020).

Collectively these findings suggest that HNO donors may be particularly useful in acute cardiovascular emergencies associated with NO• resistance. Indeed, the ability of HNO to rapidly unload the heart (venous dilation) (Paolocci et al., 2001b), improve coronary blood flow (arterial dilation) (Paolocci et al., 2003; Andrews et al., 2015) and inhibit platelet aggregation (Bermejo et al., 2005; Bullen et al., 2011b; Dautov et al., 2013) is highly advantageous following an ischemic event. Moreover, the positive inotropic and lusitropic properties of HNO (Paolocci et al., 2003; Sabbah et al., 2013) provide a unique therapeutic approach in which an improvement in myocardial performance can also be achieved in this setting. Future studies should also determine if HNO donors have the ability to stabilize mast cells and if this action is preserved in the face of impaired NO• signaling. Indeed, mast cell stabilization is critical for the prevention of plaque rupture and coronary spasm, events which trigger most cardiac ischemic emergencies (Kovanen and Bot, 2017). A caveat of many nitrovasodilators is the coronary steal phenomenon, where non-specific vasodilators induce dilation in non-ischemic regions and reduce systemic blood pressure, causing blood flow to be directed away from ischemic regions of need (Harrison and Bates, 1993). Whilst there is no current evidence that HNO donors cause coronary steal, this concept has not been fully interrogated and whether HNO selectivity targets ischemic sites remains unknown. With the recent development of the next-generation HNO donors and their ongoing clinical evaluation, these concepts will need to be investigated.

To date, the therapeutic benefits of short-term HNO administration has been a key focus. However, many of the properties of HNO confer potential for long-term use in the treatment of cardiovascular pathologies associated with impaired NO• signaling. Thus in addition to the vasodilatory, anti-aggregatory and inotropic actions of HNO donors, their ability to attenuate oxidative stress (Lin et al., 2012; Miller et al., 2013), inflammation (Andrews et al., 2016) and cardiac hypertrophy (Lin et al., 2012; Irvine et al., 2013) and their resistance to tolerance development (Irvine et al., 2011; Andrews et al., 2015) is advantageous. Indeed, the long-term cardioprotective actions of HNO in the diabetic heart is supported by our finding that chronic *in vivo* administration of the HNO donor, 1-nitrosocyclohexyl acetate (1-NCA, daily i.p. injection for 4 weeks) to streptozotocin-treated mice, attenuated left ventricular diastolic dysfunction and cardiomyocyte hypertrophy (Cao et al., 2015). With the recent development of HNO donors with more favorable pharmacokinetic properties (del Rio et al., 2014; Hartman et al., 2018), it is anticipated that the therapeutic potential of this class of compound in the treatment of both acute and chronic cardiovascular diseases will be rigorously investigated.

#### Next-Generation Nitroxyl Donors

Given the short half-life, poor aqueous solubility and active by-products released by the abovementioned HNO donors, novel synthetic pure HNO donors have now been developed. These include CXL-1020, which non-enzymatically decomposes to HNO with a half-life of approximately 2.1 min (Sabbah et al., 2013). CXL-1020 has been shown to induce positive inotropic and lusitropic effects in murine cardiomyocytes from healthy or failing hearts, and these effects were also observed *in vivo* in failing canine hearts (Sabbah et al., 2013). In patients with acute decompensated heart failure, intravenous infusion (4–6 h) of CXL-1020 enhanced cardiac function by reducing left and right ventricular pressures, decreasing systemic vascular resistance, and increasing cardiac output and stroke volume (Sabbah et al., 2013). These hemodynamic changes were not associated with alterations in heart rate, or the occurrence of arrhythmias, highlighting the safety, efficacy and potential therapeutic utility of CXL-1020 for the treatment of cardiovascular disease, where responsiveness to NO• is diminished (Sabbah et al., 2013).

These discoveries have led to the development of other HNO donors with greater tolerability and more suitable half-lives for therapeutic use in humans (Hartman et al., 2018). Of these, the HNO donor BMS-986231 (half-life; 40–144 min), has been shown to enhance cardiac contractile and relaxant responses, while promoting vasodilation and reducing myocardial oxygen consumption in canine models of heart failure (Hartman et al., 2018). Moreover, in a phase I clinical trial in healthy individuals, BMS-986231 (24- or 48-hour intravenous infusion) was well tolerated, as the only drug-related adverse event reported was the development of headaches, which were alleviated following hydration, and are a common side effect of vasodilator therapy (Cowart et al., 2019). Further, the vasodilator capacity of BMS-986231 was evident with the HNO donor causing dose-dependent reductions in systolic and diastolic blood pressure, which were sustained during infusion, and returned to baseline following infusion cessation (Cowart et al., 2019). Similar findings were also observed in patients with heart failure, where BMS-986231 reduced pulmonary arterial systolic and diastolic pressure, while decreasing total peripheral vascular resistance (Tita et al., 2017). Importantly, these hemodynamic changes were not associated with changes in heart rate or the presence of arrhythmias (Tita et al., 2017). In the StandUP-AHF study (Study Assessing Nitroxyl Donor Upon Presentation with Acute Heart Failure), patients hospitalized with heart failure with reduced ejection fraction (HF-rEF) will receive intravenous infusions of BMS-986231 at various doses or placebo for 48 h (Felker et al., 2019). The results of this multicenter, randomized, double-blind, placebo-controlled clinical trial will provide further information about the safety and tolerability of HNO donors with regard to hypotension (Felker et al., 2019).

Whilst the poor aqueous solubility of BMS-986231 limits its clinical use to intravenous administration, orally bioavailable HNO donors are on the horizon (Tita et al., 2017). CXL-1036 is an orally available HNO donor that also has a half-life (30 minutes) suitable for *in vivo* use and has been shown to enhance cardiac contraction and relaxation, and reduce myocardial demand, without altering heart rate in a canine model of heart failure (del Rio et al., 2014).

To date, much of the focus of HNO donors has been on their therapeutic potential in the treatment of acute decompensated heart failure. However, the novel vaso- and cardio-protective properties of HNO highlight the therapeutic potential of HNO donors in the treatment of a range of vascular and cardiac pathologies, particularly where NO• signaling and responsiveness is impaired, such as in T2DM. We eagerly await future studies which will examine the ability for HNO donors to overcome NO• resistance in patients with T2DM, and alleviate cardiovascular complications associated with this disease.

## Conclusion

A loss in the generation, bioavailability and responsiveness to vasoprotective NO• is a key contributor to the cardiovascular dysfunction and propensity towards acute myocardial ischemia associated with T2DM. Underpinning the impairment in NO• signaling (termed NO• resistance) is an increase in oxidative stress, driven predominantly by hyperglycemia. The impact of elevated ROS generation is far reaching, leading not only to impaired vasodilator and anti-aggregatory capacity, but ab initio reduction in therapeutic utility of NO•-based therapeutics. NO• resistance constitutes an independent risk factor for subsequent cardiovascular morbidity and mortality, and there is an urgent need to treat diabetes associated endothelial dysfunction and NO• resistance. Although perhexiline, statins and some ACE inhibitors have shown promise in their ability to improve hemodynamic and vasodilator responses in diabetes, there are limitations associated with their use in emergency treatment of cardiovascular disorders. HNO donors, however, present novel pharmacological properties, including circumvention of NO• resistance, which may facilitate a new therapeutic approach to treat diabetes-associated cardiovascular complications. 

## Author Contributions 

AV and BK-H were responsible for the design and draft of the manuscript. CQ, OW, JH, and RR provided critical review and revision of the manuscript. All authors provide approval for publication of the content.

## Funding

This work was supported in part by a National Health & Medical Research Council Project Grant (ID: APP1120859) to RR, JH, and BK-H.

## Conflict of Interest

The authors declare that the research was conducted in the absence of any commercial or financial relationships that could be construed as a potential conflict of interest.
